# Distinct CD8 T Cell Populations with Differential Exhaustion Profiles Associate with Secondary Complications in Common Variable Immunodeficiency

**DOI:** 10.1007/s10875-022-01291-9

**Published:** 2022-05-19

**Authors:** Adam Klocperk, David Friedmann, Alexandra Emilia Schlaak, Susanne Unger, Zuzana Parackova, Sigune Goldacker, Anna Sediva, Bertram Bengsch, Klaus Warnatz

**Affiliations:** 1 Center for Chronic Immunodeficiency (CCI), Medical Center – University of Freiburg, Faculty of Medicine, University of Freiburg, Freiburg, Germany; 2 Division of Immunodeficiency, Department of Rheumatology and Clinical Immunology, Medical Center – University of Freiburg, Faculty of Medicine, University of Freiburg, Freiburg, Germany; 3 Department of Immunology, 2nd Faculty of Medicine, Charles University and University Hospital in Motol, Prague, Czech Republic; 4 Faculty of Biology, University of Freiburg, Freiburg, Germany; 5 Clinic for Internal Medicine II, Medical Center – University of Freiburg, Faculty of Medicine, University of Freiburg, Freiburg, Germany; 6 Institute of Experimental Immunology, University of Zurich, Zurich, Switzerland; 7 Signaling Research Centres BIOSS and CIBSS, University of Freiburg, Freiburg, Germany; 8 German Cancer Consortium (DKTK), partner site Freiburg, Freiburg, Germany

**Keywords:** Immunodeficiency, T cells, CVID, Exhaustion, Activation, Differentiation

## Abstract

**Purpose:**

Common variable immunodeficiency (CVID) is the most frequent symptomatic primary immunodeficiency, with heterogeneous clinical presentation. Our goal was to analyze CD8 T cell homeostasis in patients with infection only CVID, compared to those additionally affected by dysregulatory and autoimmune phenomena.

**Methods:**

We used flow and mass cytometry evaluation of peripheral blood of 40 patients with CVID and 17 healthy donors.

**Results:**

CD8 T cells are skewed in patients with CVID, with loss of naïve and increase of effector memory stages, expansion of cell clusters with high functional exhaustion scores, and a highly activated population of cells with immunoregulatory features, producing IL-10. These findings correlate to clinically widely used B cell-based EURO classification. Features of exhaustion, including loss of CD127 and CD28, and expression of TIGIT and PD-1 in CD8 T cells are strongly associated with interstitial lung disease and autoimmune cytopenias, whereas CD8 T cell activation with elevated HLA-DR and CD38 expression predict non-infectious diarrhea.

**Conclusion:**

We demonstrate features of advanced differentiation, exhaustion, activation, and immunoregulatory capabilities within CD8 T cells of CVID patients. Assessment of CD8 T cell phenotype may allow risk assessment of CVID patients and provide new insights into CVID pathogenesis, including a better understanding of mechanisms underlying T cell exhaustion and regulation.

**Supplementary Information:**

The online version contains supplementary material available at 10.1007/s10875-022-01291-9.

## Introduction

Common variable immunodeficiency (CVID), the most prevalent symptomatic primary immunodeficiency, is characterized by decreased production of immunoglobulins and impaired ability of B cells to produce high affinity antigen-specific IgG antibodies after vaccination [1]. The disease is clinically highly heterogeneous, and two subgroups of patients are commonly recognized on clinical basis, those who only suffer from increased susceptibility to infections and those with a more complex disease featuring additionally phenomena of immune dysregulation, including autoimmune hemolytic anemia, interstitial lung disease (ILD), persistent non-infectious diarrhea, and others [2].

The humoral defect in CVID is associated with an altered B cell homeostasis, and the presence or absence of B cell subpopulations has been widely used for disease classification [3].

CD4 T cell studies have shown a reduction of recent thymic emigrant and naïve CD4 T cells [4, 5] and an early commitment to the follicular lineage in patients with autoimmune cytopenias [6] possibly driven by bacterial endotoxemia, which concurrently impairs CD4 T cell proliferative capacity and production of IFN-γ and IL-2 [7]. Reinforcing the notion that the cellular immunity is altered in patients with complicated form of CVID, our group previously noted a shift towards IFN-γ-producing Th1 memory and follicular helper T cells [8], a finding which extended from peripheral blood into tissues like the lung and bronchoalveolar fluid of CVID patients with ILD [9] and the gastrointestinal tract [10]. A recent study has also shown higher expression of inhibitory receptors PD-1, LAG3, CTLA-4, and TIGIT in memory CD4 T cells of patients with complex CVID, which nevertheless retained capacity to produce IFN-γ and proliferate [11].

The CD8 compartment in CVID remains less explored, with studies reporting normal [4, 12], decreased [13], or increased [14] absolute CD8 T cell counts, but unanimously describing higher proportion of overall CD8 T cells, with low proportions of naïve, but elevated effector memory CD8 T cells [15, 16] and terminal effector cells expressing CD57 and KLRG1 [17], altered response to TLR stimulation [18], substantial activation measured by the expression of HLA-DR and CD38 [19–22], and increased expression of cytotoxic markers such as granzyme B [19] in patients with autoimmune complications and lymphoproliferation. CD8 T cells are involved in the pathogenesis of some manifestations such as enteropathy [23], hepatopathy [24], and potentially even in ITP [25]. Prolonged stimulation by both autoantigens and external pathogens may ultimately result in chronic activation and ongoing inflammation, accelerating CD8 T cell differentiation and possibly leading to features of CD8 T cell exhaustion described in detail in other diseases with chronic inflammation [26, 27]. However, the role of T cell exhaustion in CVID remains unclear.

Thus, we set out to utilize recent advances in understanding of CD8 T cell biology, especially with focus on their differentiation, exhaustion, and progenitor capacity, using highly multiplexed immune phenotyping and unsupervised exploratory approaches in a large cohort of genetically diverse patients with infection only and complicated CVID. A challenge in profiling T cell exhaustion is the overlap of exhaustion markers with effector or memory T cell populations. We therefore used a multifaceted exhaustion profiling approach that focuses on analysis of markers highly biased to expression in exhausted T cells in combination with a high-dimensional single-cell phenotyping approach as well as functional assays to profile cytokine co-expression patterns typical for exhausted T cells [28, 29]. Our work points to the expansion of exhausted CD8 T cells with immunoregulatory features particularly in CVID patients with complex disease manifestation, suggesting them as possible contributors to disease activity but also highlighting them as potential biomarkers for high-risk patients.

## Methods

### Patients and Samples

Peripheral blood was obtained from patients with CVID seen at the immunodeficiency clinic of the Freiburg University Medical Center, Freiburg, Germany. All patients fulfilled the criteria for CVID according to the European Society for Immunodeficiencies (www.esid.org). The following clinical data were recorded: splenomegaly (defined as a diameter of > 11 × 4 × 7 cm, as shown using ultrasonography or computed tomography [CT]); generalized lymphadenopathy (lymph nodes > 1 cm in diameter in ≥ 2 different anatomic sites detected by means of clinical examination, ultrasonography, or CT); granulomatous disease (suggested by CT or proven by histology); autoimmune cytopenias (autoimmune hemolytic anemia or thrombocytopenia); interstitial lung disease (based on CT morphology and bronchoalveolar lavage or biopsy); hepatopathy (based on ultrasound, elastography, duplex, laboratory parameters); and enteropathy (exclusion of infection and histology). Patients with only splenomegaly or lymphadenopathy were classified as “infection only” (CVIDio), whereas patients with any of the other complications were classified as “complex disease” (CVIDc). Patients who never underwent the respective diagnostic workup were excluded from the analysis. Patients were further classified according to EUROclass classification, based on the reduction of switched memory B (smB) cells and the expansion of CD21low B cells. Description of patient cohort can be inspected in Table 1.

**Table 1:** Cohort description

ID	Category	Genetic cause	Freiburg classification	EUROclass classification	Sex	Splenomegaly	LAD	Lymphoma	AIC	Diarrhea	Norovirus	ILD	Granulomatous disease	Hepatopathy	Therapy
1	CVIDc	CTLA-4	Ia	B + smB + 21lowTrnorm	F	1	1	1	1	1	0	1	0	0	Budesonide
2	CVIDc	NFkB1	Ib	B + smB-21lowTrnorm	F	1	1	0	1	1	0	1	0	1	no IS
3	CVIDc	Unknown	Ia	B + smB-21lowTrhigh	F	1	1	0	0	0	NA	1	1	0	Prednisolone, abatacept
4	CVIDc	NA	Ib	B + smB-21normTrnorm	F	1	0	0	1	0	NA	0	0	0	Prednisolone, abatacept
5	CVIDc	NA	Ib	B + smB-21lowTrnorm	M	1	0	0	0	0	NA	1	0	0	Prednisolone, budesonide
6	CVIDc	Unknown	Ia	B + smB-21lowTrnorm	M	1	0	0	0	1	1	0	0	0	Budesonide
7	CVIDc	Unknown	Ib	B + smB-21lowTrnorm	F	1	1	0	0	0	NA	1	1	0	no IS
8	CVIDc	Unknown	Ia	B + smB-21lowTrhigh	M	1	1	0	1	1	0	0	1	0	no IS
9	CVIDc	Unknown	Ib	B + smB-21lowTrhigh	F	1	1	0	1	1	0	1	1	0	no IS
10	CVIDc	Unknown	Ia	B + smB-21lowTrnorm	F	1	1	0	1	1	0	1	1	0	no IS
11	CVIDc	CTLA-4	Ia	B + smB-21lowTrnorm	M	1	0	1	1	1	0	0	0	0	Prednisolone, budesonide
12	CVIDc	NFkB2	Ib	B + smB-21normTrhigh	F	0	0	0	0	0	NA	0	0	1	no IS
13	CVIDc	NFkB1 + TACI l.b	Ia	B + smB + 21lowTrnorm	M	0	0	0	0	1	NA	0	0	1	no IS
14	CVIDc	Unknown	Ib	B + smB-21normTrnorm	F	1	0	0	0	0	NA	0	1	0	Prednisolone, sulfasalazine
15	CVIDc	Unknown	Ia	B + smB-21lowTrnorm	F	1	1	0	0	1	0	1	0	1	no IS
16	CVIDc	Fas	Ib	B + smB-21normTrhigh	F	1	0	0	0	1	0	0	0	0	Budesonide
17	CVIDc	NA	Ib	B + smB-21lowTrnorm	F	1	0	0	0	1	NA	1	0	0	no IS
18	CVIDc	TACI l.b	Ia	B + smB-21lowTrnorm	M	1	1	0	1	1	NA	1	0	0	no IS
19	CVIDc	Unknown	Ib	B + smB-21normTrnorm	M	1	0	0	0	1	0	1	0	0	no IS
20	CVIDc	Unknown	II	B + smB + 21lowTrnorm	F	1	1	0	1	1	0	1	1	0	no IS
21	CVIDc	Unknown	Ia	B + smB-21lowTrnorm	F	1	1	0	0	1	0	1	1	1	no IS
22	CVIDc	Unknown	Ia	B + smB + 21lowTrnorm	M	1	1	0	1	0	NA	1	0	0	Prednisolone, budesonide, abatacept
23	CVIDc	Unknown	Ia	B + smB + 21lowTrnorm	M	1	1	0	1	0	NA	0	1	1	no IS
24	CVIDc	Unknown	Ia	B + smB-21lowTrhigh	F	1	1	0	1	1	1	1	1	1	Budesonide
25	CVIDc	Unknown	II	B + smB + 21normTrnorm	F	1	0	0	0	1	0	1	1	0	no IS
26	CVIDc	VUS in SOCS1	Ia	B-, previously B + smB-21low	F	1	0	0	1	1	NA	0	0	0	Prednisolone, post RTX
27	CVIDc	Unknown	Ib	B + smB-21lowTrnorm	M	1	1	0	0	1	0	0	0	0	no IS
28	CVIDc	NFkB1	Ib	B + smB-21normTrnorm	M	0	1	0	0	1	1	0	0	1	no IS
29	CVIDc	LRBA	Ia	B-	M	1	0	0	1	1	0	1	0	0	Prednisolone, budesonide
30	CVIDc	Unknown	II	B + smB + 21normTrnorm	M	1	0	0	0	0	NA	0	0	1	Prednisolone
31	CVIDio	Unknown	Ib	B + smB-21normTrnorm	M	1	0	0	0	0	NA	0	0	0	no IS
32	CVIDio	Unknown	Ib	B + smB-21normTrnorm	F	0	0	0	0	0	NA	0	0	0	no IS
33	CVIDio	NA	Ia	B + smB + 21normTrhigh	M	0	0	0	0	0	NA	0	0	0	no IS
34	CVIDio	NFkB1	Ia	B + smB-21lowTrnorm	M	0	0	0	0	0	NA	0	0	0	no IS
35	CVIDio	Unknown	Ia	B-	M	1	0	0	0	0	NA	0	0	0	no IS
36	CVIDio	BAFFR	Ib	B + smB + 21normTrhigh	M	0	0	0	0	0	0	0	0	0	no IS
37	CVIDio	Unknown	Ib	B + smB-21normTrhigh	M	1	0	0	0	0	NA	0	0	0	no IS
38	CVIDio	Unknown	Ib	B + smB + 21normTrnorm	M	0	0	0	0	0	NA	0	0	0	no IS
39	CVIDio	Unknown	Ib	B + smB-21normTrnorm	F	1	0	0	0	0	0	0	0	0	no IS
40	CVIDio	Unknown	Ib	B + smB-21normTrnorm	F	0	0	0	0	0	NA	0	0	0	no IS

*AIC*, autoimmune cytopenia; *CVIDc*, complex CVID; *CVIDio*, infection only CVID; *ILD*, interstitial lung disease; *LAD*, lymphadenopathy; *l.b.*, likely benign; *NA*, not available; *no IS*, no immunosuppression; *RTX*, rituximab; *Unknown*, whole exome or panel sequencing performed, but no hits explaining phenotype were found; *VUS*, variant of unknown significance

### Flow Cytometry

Following peripheral venipuncture, peripheral blood mononuclear cells (PBMCs) were isolated from ethylenediaminetetraacetic acid (EDTA)-anticoagulated blood by centrifugation on a density gradient Pancoll (PAN Biotech, Aidenbach, Germany) and then frozen at − 180 °C in 10% dimethyl sulfoxide (DMSO) and 20% fetal bovine serum (FBS). Samples were thawed in batches, washed 2 times with RPMI 1640 (Pan Biotec, Aidenbach) + 10% FCS (Merck, Darmstadt, Germany) + 1% PenStrep (Life Technologies, Darmstadt, Germany), and labeled with respective antibodies for detection of indicated cell surface molecules. When applicable, fixation and permeabilization were performed with the eBioscience Intracellular Fixation & Permeabilization Buffer Set (ThermoFisher Scientific, Darmstadt, Germany). Data was acquired on LSR II flow cytometer (BD Biosciences, Franklin Lakes, NJ) and analyzed with FlowJo software (version 7.6.5 or 10.5.3; TreeStar, Ashland, Ore). List of used antibodies can be viewed in Table S1. The gating strategy is shown in Figure S1.

### Mass Cytometry

For mass cytometry, 2 million viable PBMCs per patient (4 HD, 4 CVIDio, 4 CVIDc) were stimulated with 0.05 μg/ml phorbol 12-myristate 13-acetate (PMA) and 1 μg/ml ionomycin for 5 h, with 0.5 μg/ml GolgiPlug and 0.032 μg/ml GolgiStop added 1 h into the stimulation. Cells were then stained for mass cytometry. Mass cytometry reagents were obtained or generated by custom conjugation to isotope-loaded polymers using the Maxpar X8 antibody labeling kit. Mass cytometry antibodies used are shown in Table S2. Staining was performed after β2-microglobulin-based sample barcoding. Briefly, single-cell suspensions were pelleted, incubated with 20 mM Lanthanum-139-loaded maleimido-mono-amine-DOTA in PBS for 5 min at RT for live/dead discrimination (LD). Cells were washed in staining buffer and resuspended in surface antibody cocktail, incubated for 30 min at RT, washed twice in staining buffer, fixed and permeabilized using FoxP3 staining buffer set (ThermoFisher Scientific), and stained intracellularly for 60 min at RT. Cells were further washed twice before fixation in 4% PFA solution containing 125 nM Iridium overnight at 4C. Prior to data acquisition on CyTOF Helios (Fluidigm), cells were washed twice in PBS and once in cell acquisition solution. After acquisition, all CyTOF files were normalized together using the Bead-based Nolan Lab normalizer (available from https://github.com/nolanlab/bead-normalization/releases). Afterwards, clean-up of the FCS files was performed with FlowJoV10 by using Gaussian-derived parameters “residual,” center,” “offset,” “width,” “event length,” MM-Dota, and iridium to eliminate dead cells, debris, and normalization beads. Files were concatenated using FlowJoV10 prior to manual debarcoding. For data analysis, including Wanderlust [30], FlowSOM [31], and UMAP [32], the online data analysis platform OMIQ (www.omiq.ai) was used (Omiq, Santa Clara, CA). For Wanderlust, FlowSOM, and UMAP, pseudotime, clustering, and dimensionality reduction were calculated based on the following markers: 2B4, CCR7, CD7, CD8, CD16, CD27, CD28, CD38, CD39, CD45RA, CD57, CD73, CD127, CD120b, CD160, CTLA-4, CXCR5, Eomes, FOXO1, FoxP3, Helios, HLA-DR, LAG-3, PD-1, Tbet, TCF1, TIGIT, Tim3, and TOX. A functional exhaustion score (FES) was calculated using the production of IL-2 and XCL-1, as well as IFN-γ and TNF-α coproduction (2*(%IFN-γ^+^TNF-α^−^)-(% IFN-γ^−^ TNF-α^+^)-(%IL-2^+^))*(%XCL-1^+^) [28].

### Study Approval

Written informed consent with participation in this study was obtained from all patients and control subjects in accordance with the ethical standards of the institutional and national research committee and with the 1964 Helsinki Declaration and its later amendments. The study was approved by local authorities (University of Freiburg Ethics Committee 239/1999 and 121/11).

### Statistical Analysis

In the boxplots used throughout the manuscript, boxes depict the 25th and 75th percentiles (first and third quartile, respectively), and whiskers depict the 2.5–97.5th percentiles. In violin plots, medium smoothing was used, and the plots span from the lowest to highest measurement, with median and 1st and 3rd quartiles shown. Tests used are specified in each results section.

**Fig. 1:**
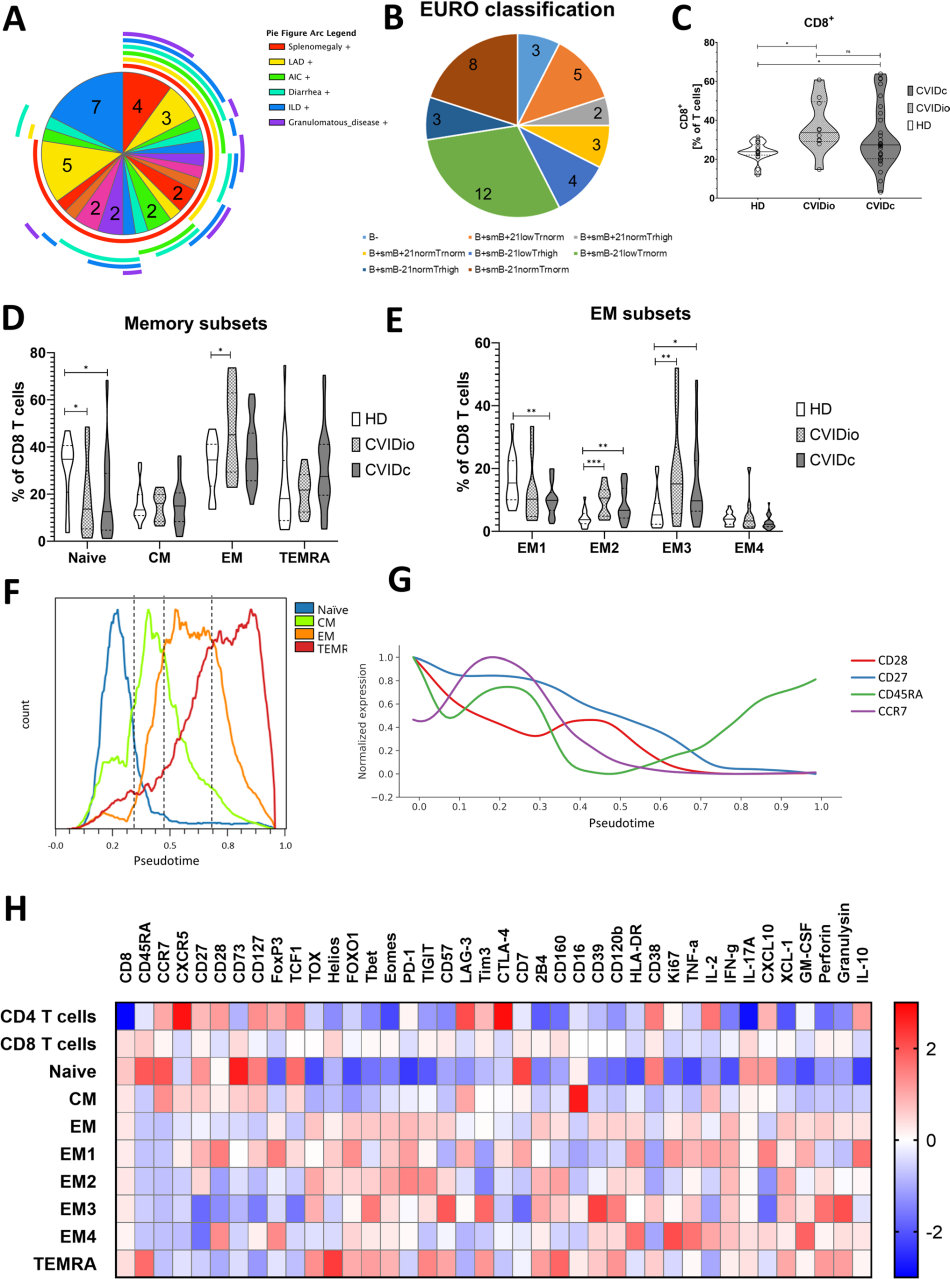
Clinical features of the cohort and changes in CD8 differentiation stages. **A** Complications seen in the studied cohort of CVID patients (*n* = 40, 11 CVIDio and 29 CVIDc). **B** EURO classification of the studied cohort. **C** Proportion of CD8 T cells in HD, CVIDio, and CVIDc. **D**, **E** Differentiation stages of CD8 T cells assessed by flow cytometry. **F** Distribution of differentiation stages in Wanderlust-derived pseudotime on 12 samples (4 HD, 4 CVIDio, 4 CVIDc) assessed by CyTOF. **G** Changes in basic differentiation markers over Wanderlust-derived pseudotime. **H** Heatmap showing phenotype of PBMCs from 4 HD, 4 CVIDio, and 4 CVIDc after stimulation with PMA and ionomycin, assessed by CyTOF. Expression normalized per marker, color scale corresponds to *Z* values

Statistical analyses and the generation of graphs were performed in the statistical language and environment R, version 3.6.3; GraphPad Prism software (version 8.0.1; GraphPad Software, San Diego, CA, USA); and Microsoft Excel 2016 (Microsoft, Redmond, WA, USA). The Simplified Presentation of Incredibly Complex Evaluations (SPICE) plots shown in Fig. 1 were constructed using the SPICE 6 software [33].

## Results

### Relative Expansion of CD8 T Cells in CVID Comprises Mainly Increased Percentages of EM2 and EM3 Subpopulations

Thirty adult patients with CVIDc (17 female, 13 male), 10 adult patients with CVIDio (3 female, 7 male), and 17 healthy adults with no history of immune disease were included in this study. Detailed description of the cohort, including genetic findings and Freiburg and EUROclass classifications, can be found in Table 1. The most common clinical complications were recurrent non-infectious diarrhea (*n* = 21), interstitial lung disease (ILD) (*n* = 17), and lymphadenopathy (LAD) (*n* = 16), followed by autoimmune cytopenia (AIC) (*n* = 14). Splenomegaly was present in 31 patients but when found in isolation with no other clinical complications did not qualify the patient as CVIDc. A graph showing co-incidence of various clinical features in the studied cohort can be found in Fig. 1A, and the proportion of patients in the different EUROclass phenotypes can be found in Fig. 1B.

We next performed a detailed analysis of the differentiation and exhaustion of CD8 T cells in CVID patients. Patients with CVID had higher proportion of CD8 T cells than healthy controls (HD) in the peripheral blood (Fig. 1C) (Welch’s ANOVA *p* = 0.008, unpaired *t* tests with Welch’s correction) (CD4:8 ratio in Figure S2), with a shift from naïve (CD45RA^+^CCR7^+^) towards effector memory (EM, CD45RA^−^CCR7^−^) and terminal effector memory cells (TEMRA, CD45RA^+^CCR7^−^) stages (Fig. 1D). In particular, EM2 (CD27^+^28^−^) and EM3 (CD27^−^28^−^) cells were significantly expanded in both CVIDio and CVIDc compared to HD (Fig. 1E) (CVIDio EM2 unpaired *t* test with Welch’s correction *p* < 0.001, EM3 *p* = 0.007, CVIDc EM2 *p* = 0.002, EM3 *p* = 0.01).

For detailed exhaustion profiling, where phenotyping of T cells based on differentiation markers is insufficient to precisely identify exhausted T cell populations, we combined a highly multiplexed phenotyping of exhaustion-biased immune markers with a functional analysis of the expression patterns of (*n* = 10) cytokines important for exhausted T cell biology. This advanced approach allows for the identification of relevant exhausted T cell phenotypes and evaluation of an exhaustion-typical dysfunction pattern (i.e., reduced IL-2 and TNF-α production but possible production of IFN-γ and XCL-1 chemokine) that can be assessed on a single-cell level using a functional exhaustion score, as previously published [28]. For this reason, we stimulated PBMCs from 4 healthy donors, 4 CVIDio (patient ID 31, 33, 38, 40) and 4 CVIDc (patient ID 5, 10, 17, 23) patients with PMA + ionomycin and analyzed them by using a mass cytometry panel of 44 extracellular markers, transcription factors, cytokines, and chemokines (for a complete list of markers, see Table S2).

To assess if the analysis of the pre-defined canonical T cell subsets correlated with data-driven approaches, we then performed trajectory-inference analysis using Wanderlust [30] based on 29 phenotypic markers (for a list see Methods). The resulting pseudotime variable corresponded well with these canonical differentiation stages from naïve into central, effector memory, and ultimately TEMRA populations (Fig. 1F, G), documenting expected changes with gradual decrease and final re-expression of CD45RA, loss of CCR7, preserved in CM, and loss of CD27 and CD28.

When stimulated with PMA and ionomycin, the EM2 and EM3 CD8 T cells had a unique phenotype which on average had decreased production of IL-2 but high production of cytotoxic molecules perforin, granulysin, and the lymphotactin XCL-1 (Fig. 1H, dotplots in Figure S3). Especially EM3 cells also displayed a high expression of the senescence marker CD57, increased in chronic immune activation [34], the exhaustion marker Tim3, the ectonucleotidase CD39, and the TNF-α receptor CD120b. Together, this data revealed significant heterogeneity of exhaustion markers and functional T cell profiles in canonical T cell differentiation subsets.

### CD8 T Cell Landscape Is Grossly Disturbed in CVID, with Enriched Activated and Exhausted Cellular Populations and Cells with Immunoregulatory Potential But Lacking Progenitor Capability

**Fig. 2:**
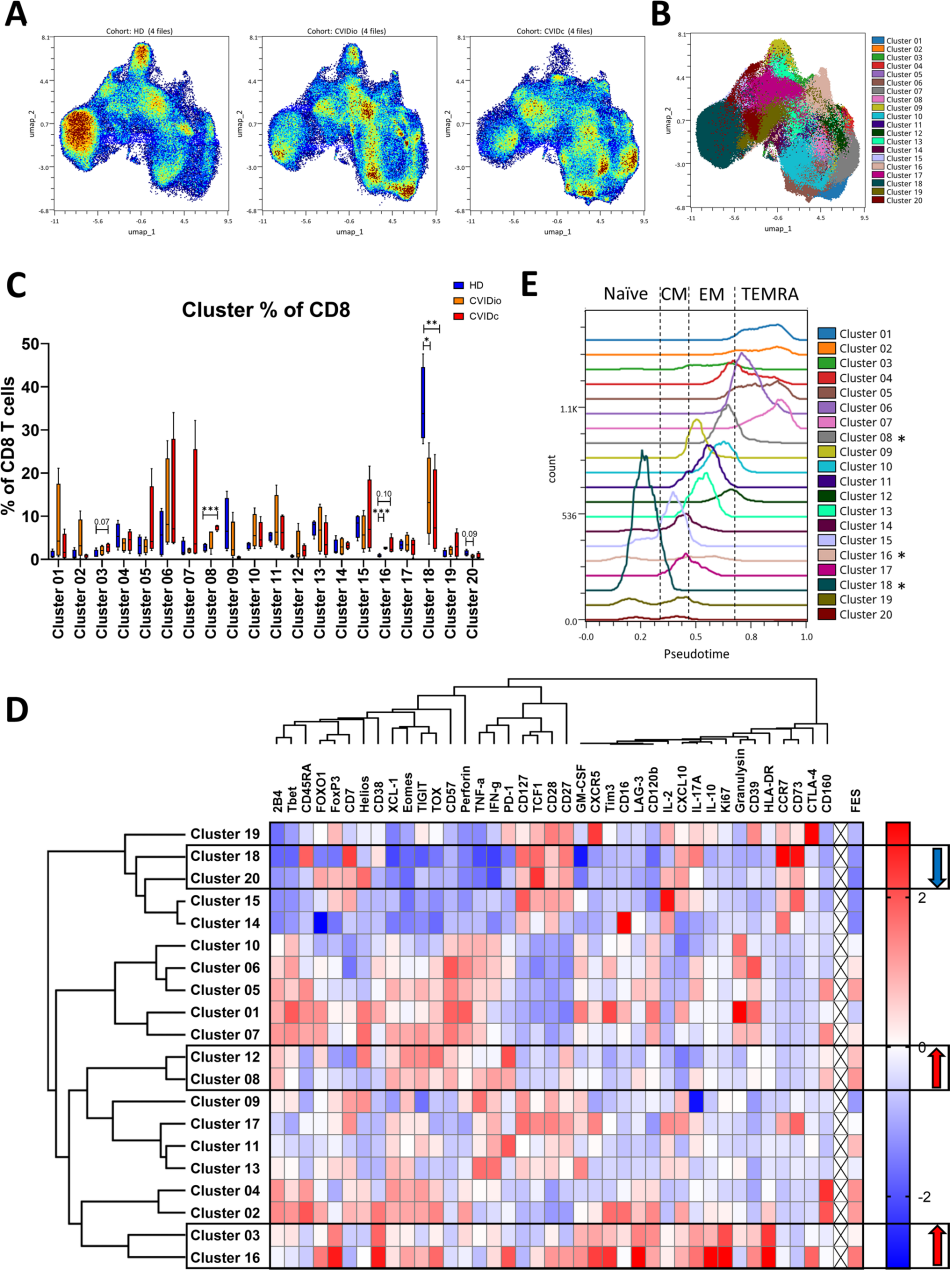
Multiparametric analysis of the CD8 compartment as assessed by CyTOF (*n* = 12, 4 HD, 4 CVIDio, and 4 CVIDc). **A** UMAP visualization of CD8 T cells in concatenated HD, CVIDio, and CVIDc cohorts. **B** FlowSOM clusters displayed in UMAP dimensions. **C** FlowSOM cluster proportions in the three cohorts. **D** FlowSOM cluster phenotype heatmap, with calculated FES. **E** Cluster distribution in Wanderlust pseudotime as corresponds with canonical naïve, CM, EM, and TEMRA populations, with clusters significantly affected in CVID marked with an asterisk

To understand the exhaustion programs of CD8 T cells in CVID in more detail, we analyzed the high-dimensional dataset in a data-driven, unbiased manner. Dimensionality reduction on 29 phenotypic markers was performed by UMAP, revealing major differences between HD, CVIDio, and CVIDc cohorts (Fig. 2A). Clustering of high-dimensional data by self-organizing maps algorithm (FlowSOM) revealed 20 distinct cell clusters (optimal number of clusters selected using the elbow method) (Fig. 2B), the proportion of which was significantly different between the three cohorts (Fig. 2C, Figure S4). Cluster 8 was significantly expanded in CVIDc compared to HD (unpaired *t* test with Welch’s correction *p* = 0.0002); cluster 16 was significantly expanded in CVIDio compared to HD (*p* = 0.0002, CVIDc × HD *p* = 0.1), whereas cluster 18 was significantly diminished in both CVIDio and CVIDc compared to HD (CVIDio × HD *p* = 0.01, CVIDc × HD *p* = 0.009). Several other clusters (cluster 3, 6, 10, 12) were insignificantly expanded in both CVID cohorts. The clusters provided higher granularity compared to the canonical differentiation stages shown in Fig. 1 and showed different localization across the canonical developmental pseudotime (Fig. 2E).

We then compared the expression of all phenotypic and functional markers across all 20 clusters (Fig. 2D). Further, we calculated a functional exhaustion score (FES) as described previously to identify clusters with a polyfunctionality pattern typical for exhausted T cells [28], based on TNF-α, IFN-γ, IL-2, and XCL-1 production. FES was mediocre or high in clusters expanded in CVIDc, with a similar trend in CVIDio, and low in clusters more prevalent in healthy donors, and it was significantly different between clusters (Welch’s ANOVA *p* < 0.0001) (Figure S5). These findings link clusters with an exhaustion phenotype and FES to CVID, in particular in CVIDc patients.

Clusters 3 and 16, which were upregulated in CVID, displayed strong expression of the activation markers CD38 and HLA-DR, the proliferation-associated nuclear protein Ki67, and produced IL-10 and IL-17A, to a lesser extent also the effector cytokine IFN-γ, but little or none TNF-α as well as no perforin and granulysin. Their expression patterns then diverged, with cluster 3 having low expression of most exhaustion-associated markers, whereas cluster 16 displayed an elevated proportion of cells staining positive for CXCR5; higher expression levels of transcription factors TOX, FOXO1, Helios, and Eomes; and inhibitory molecules such as PD-1, TIGIT, LAG3, Tim3, and CTLA-4 and had a particularly high FES. The phenotype of cells in cluster 16 is reminiscent of the elusive regulatory CD8 T cell population, based on their production of IL-10, PD-1 expression, and higher average expression of the transcription factor FoxP3 in comparison to other CD8 T cells (Figure S6) [35, 36].

Cluster 6 bore the hallmarks of senescent cells expressing CD57 and effector capabilities.

Cluster 8, also expanded in CVIDc, displayed an above-average expression of exhaustion-associated phenotypic markers Eomes, PD-1, TIGIT, LAG-3, 2B4, and CD160, with low Tbet and TCF1, suggesting terminal differentiation. These cells retained their capacity to produce TNF-α, IFN-γ, and XCL-1 but did not produce large amounts of cytotoxic molecules such as granulysin or perforin and unlike cluster 16 were not activated or proliferating. These cells together with cluster 12, which had very high expression of PD-1, fulfil most canonical signs of being exhausted and have a correspondingly high FES.

Cluster 18, severely diminished in CVID, had the hallmarks of naïve CD8 T cells, including expression of CD45RA, CCR7, CD27, CD28, CD73, CD127, and CD7.

Cluster 20, a small population but seemingly lower in CVID compared to HD, clustered together with the naïve CD8 T cells of cluster 18 but was notable for its high expression of TCF1 and FOXO1, transcription factors associated with progenitor capabilities of effector cells, in conjunction with CCR7. Similarly, cluster 19 also expressed TCF1, but also high levels of CXCR5 intermediate levels of PD-1 and no TOX, which bears similarity to progenitor population of exhausted T cells [37, 38]. Cluster 19 was comparably prevalent in both CVID patients and HD.

Thus, the CyTOF analysis revealed the reduction of naïve CD8 T cells and CCR7^+^TCF1^+^FOXO1^+^ stem cell-like progenitors but not exhaustion-specific CXCR5^+^PD-1^int^TCF1^+^ progenitors. In contrary, two distinct groups of cells were more prevalent in CVID, a group of highly differentiated classical exhausted cells with high FES lacking signs of ongoing activation and production of cytokines and a group of highly activated and proliferating cells with immunoregulatory features such as the production of IL-10 and expression of CXCR5 and higher average expression of FoxP3.

### Bulk CVID CD8 T Cells Show Features of Exhaustion, Activation, and Cytotoxicity

**Fig. 3:**
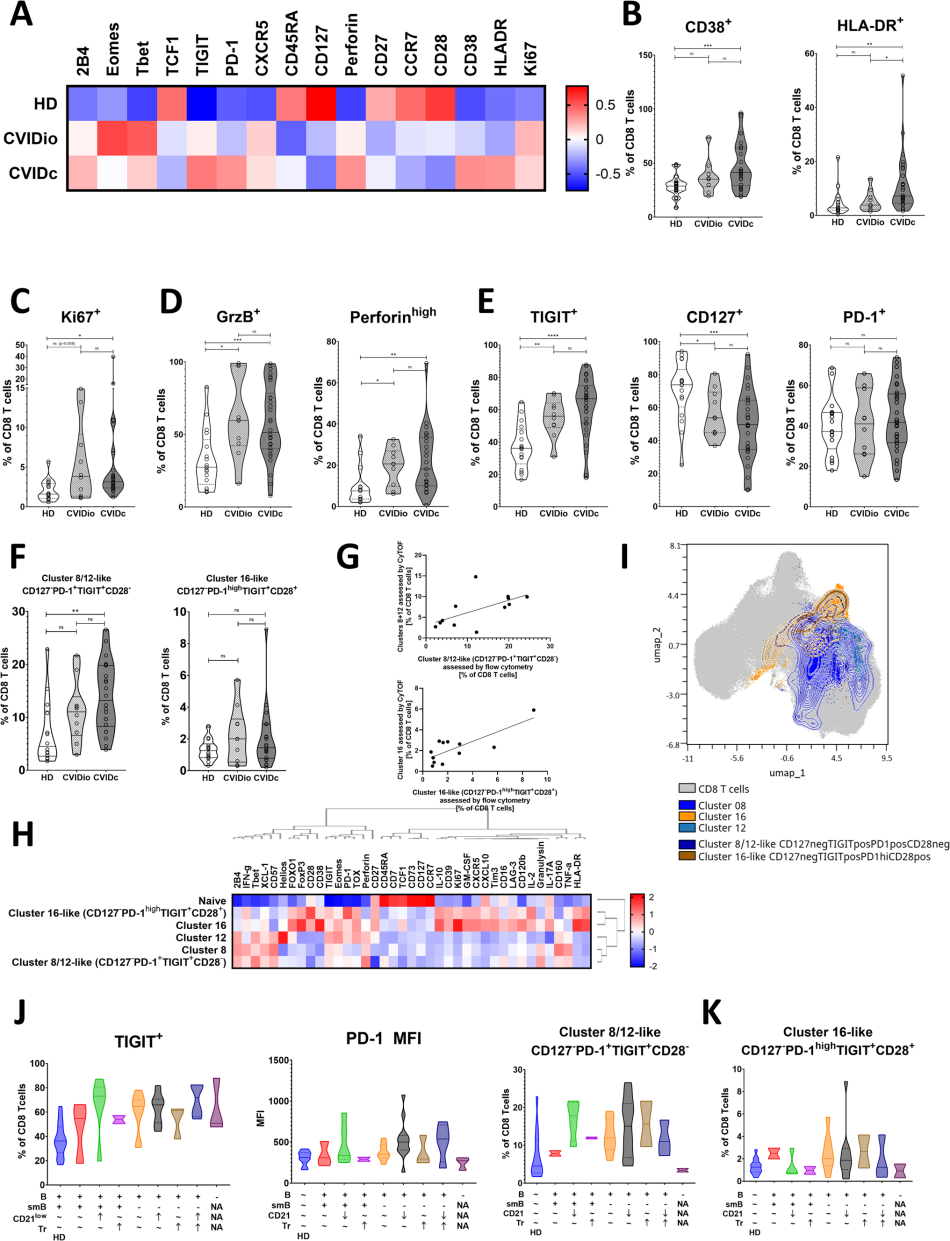
Flow cytometric evaluation of single markers and CyTOF-like clusters in a larger cohort (*n* = 57, 17 HD, 11 CVIDio, and 29 CVIDc). **A** Heatmap showing expression levels of single markers on bulk CD8 T cells in HD, CVIDio, and CVIDc (*n* = 57). **B** Activation, **C** proliferation, **D** cytotoxicity, and **E** differentiation and exhaustion markers on bulk CD8 T cells in HD, CVIDio, and CVIDc. **F** Prevalence of CD127^−^PD-1^+^TIGIT^+^CD28^−^ (cluster 8/12-like) and CD127^−^PD-1^high^TIGIT^+^CD28^+^ (cluster 16-like) cells assessed by flow cytometry (*n* = 32) in HD, CVIDio, and CVIDc. **G** Correlation between cluster 8/12-like and cluster 16-like populations assessed by flow cytometry and cluster 8 + 12 and cluster 16 cells assessed by CyTOF in identical samples (*n* = 12). **H** Heatmap showing expression of 39 markers assessed by CyTOF in clusters 8, 12, and 16, cluster 8/12-like, cluster 16-like, and naïve (CD45RA^+^CCR7^+^) cells (*n* = 12, 4 HD, 4 CVIDio, and 4 CVIDc). **I** Co-localization of clusters 8, 12, and 16 and cluster 8/12-like and cluster 16-like cells assessed by CyTOF in UMAP dimensionality reduction space (*n* = 12, 4 HD, 4 CVIDio, and 4 CVIDc). **J**, **K** Correlation of CD8 phenotype assessed by flow cytometry with the EURO classification (*n* = 57)

Following the markers of interest discovered by unbiased clustering approach shown above in a smaller cohort assessed by mass cytometry, we verified the expression pattern of exhaustion-associated markers PD-1, TIGIT, CD127, 2B4, Tbet, Eomes, and TCF1, activation markers CD38 and HLA-DR, nuclear protein Ki67, and the cytotoxicity markers perforin and granzyme B across several flow cytometric panels in the larger cohort of 40 patients and 17 healthy donors (Figs. 3A and S7).

On average, bulk CVIDc-derived CD8 T cells were activated (Fig. 3B), proliferating (Fig. 3C) and functionally active by producing cytotoxic molecules (Fig. 3D), corresponding to the expanded clusters 3, 8, 12, and 16. These features were generally more pronounced in CVIDc-derived CD8 T cells compared to CVIDio, although the difference only reached significance for the percentage of activated HLA-DR^+^ cells. CVID CD8 T cells on average had higher expression of the exhaustion-associated marker TIGIT and lost the expression of the IL-7 receptor CD127, while the proportion of PD-1^+^ cells was comparable between HD, CVIDio, and CVIDc (Fig. 3E). These changes did not merely reflect changes in the distribution of the different memory stages shown in Fig. 1 but were also present within the distinct naïve and EM subpopulations (Figure S8).

To assess cells corresponding to the dysregulated clusters seen in CyTOF analysis using the larger cohort of 40 patients and 17 healthy donors stained with flow cytometry, we gated on CD127^−^PD-1^+^TIGIT^+^CD28^−^ cells (phenotype equivalent of clusters 8 and 12, hereafter referred to also as cluster 8/12-like cells) and CD127^−^PD-1^high^TIGIT^+^CD28^+^ cells (phenotype equivalent of cluster 16, hereafter referred to also as cluster 16-like cells). Cluster 8/12-like cells were significantly more prevalent in CVIDc compared to HD, with a similar but insignificant trend for cluster 16-like cells (Fig. 3F). The prevalence of cluster 8/12-like and cluster 16-like cells determined by flow cytometry correlated to CyTOF-determined clusters 8 + 12 and cluster 16 cells in those patients assessed by both methods (*n* = 12) (Fig. 3G) (Pearson correlation *p* = 0.04 and 0.0024, *R*^2^ = 0.354 and 0.618, respectively).

Retrospectively re-assessing the CyTOF data of 4 HD, 4 CVIDio, and 4 CVIDc patients, we verified that the expression pattern of CD127^−^PD-1^+^TIGIT^+^CD28^−^ cells mirrored that of clusters 8 and 12 and the expression pattern of CD127^−^PD-1^high^TIGIT^+^CD28^+^ mirrored that of cluster 16 (Fig. 3H), and when overlaid on the UMAP visualization, these populations overlapped with their respective clusters (Fig. 3I). Functionally, CD127^−^PD-1^high^TIGIT^+^CD28^+^ expressed high levels of Ki67, IL-10, and IL-2 but unlike their CD127^−^PD-1^+^TIGIT^+^CD28^−^ counterparts lacked the cytotoxic effectors perforin and granulysin.

The expansion of TIGIT^+^ CD8 T cells was strongly associated with the EURO classification based on B cell phenotyping (see Methods and [3]) (one-way ANOVA *p* = 0.002) (Fig. 3J), as was the expression of PD-1 (one-way ANOVA *p* = 0.035) and crucially so were the exhausted cluster 8/12-like cells (CD127^−^PD-1^int^TIGIT^+^CD28^−^) (one-way ANOVA *p* = 0.0429). The association between the EURO classification and immunoregulatory cluster 16-like cells (CD127^−^PD-1^high^TIGIT^+^CD28^+^) did not reach significance (one-way ANOVA *p* = 0.58) (Fig. 3K).

These results show activation and elevated expression of exhaustion-associated molecules on bulk CD8 T cells in CVID patients. Further, they demonstrate a surrogate gating strategy to assess exhausted cells corresponding to clusters 8 and 12 and immunoregulatory cells corresponding to cluster 16 using low-parametric flow cytometry and show that these exhausted cells and markers are associated with the EURO classification system of CVID.

### Clinical Complications of CVID Such as Interstitial Lung Disease and Autoimmune Cytopenia Strongly Associate with More Pronounced Features of Exhaustion, Whereas Diarrhea Associates with T Cell Activation

**Fig. 4:**
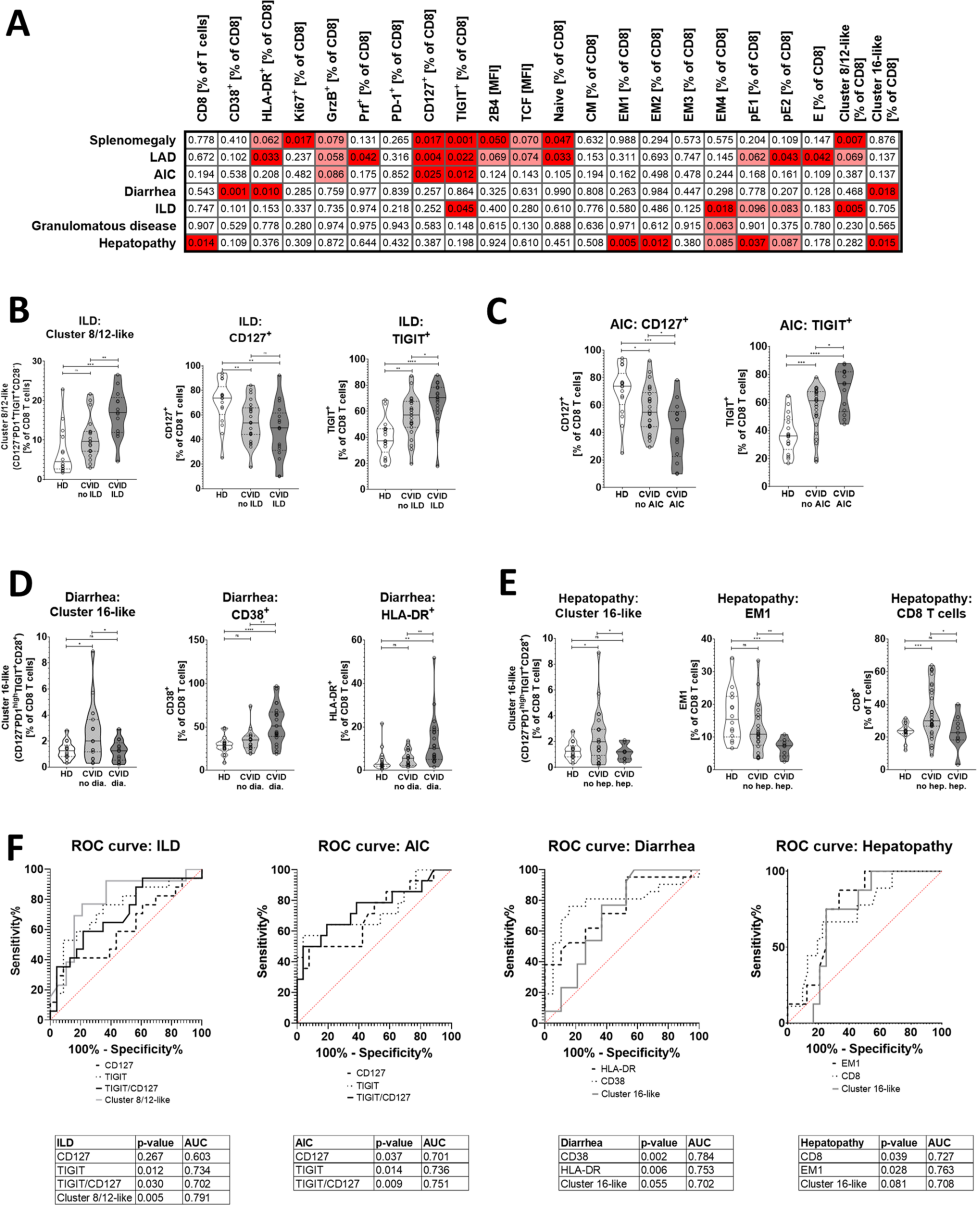
Clinical feature prediction in large cohort of CVID patients assessed by flow cytometry (*n* = 57, 17 HD, 11 CVIDio, and 29 CVIDc). **A** *p* values of main CD8 features as differentiating factors for individual clinical complications. **B** Cluster 8/12-like, CD127^+^, and TIGIT^+^ CD8 T cells in patients with/without ILD. **C** CD127^+^ and TIGIT^+^ CD8 T cells in patients with/without AIC. **D** Cluster 16-like, CD38^+^, and HLA-DR^+^ CD8 T cells in patients with/without diarrhea. **E** Cluster 16-like, EM1, and bulk CD8 T cells in patients with/without hepatopathy. **F** Receiver operating (ROC) curves for cluster 8/12-like, cluster 16-like, CD127^+^, TIGIT^+^, CD38^+^, HLA-DR^+^, and bulk CD8 T cells and the presence/absence of ILD, AIC, diarrhea, and hepatopathy

As the clinical phenotype of CVIDc is varied and patients can exhibit many different complications, we tested the association between CD8 T cell phenotype and individual clinical features seen in patients with complex disease. Figure 4A shows a table with unpaired student’s *t* test with Welch’s correction non-adjusted *p* values calculated between all CVID patients with and without each symptom for select easy-to-measure parameters, exhausted cluster 8/12-like, and immunoregulatory cluster 16-like cells. Whereas the expression of inhibitory receptors, loss of CD127, and the exhausted cluster 8/12-like cells were significantly associated with AIC, lymphadenopathy, and ILD, recurrent non-infectious diarrhea was most strongly associated with CD8 T cell activation and the activated immunoregulatory cluster 16-like cells.

ILD was strongly associated with the proportion of cluster 8/12-like cells, loss of CD127, and expression of TIGIT (Fig. 4B). Cluster 8/12-like (CD127^−^PD-1^+^TIGIT^+^CD28^−^) cells offered best combination of sensitivity and specificity when searching for ILD in CVID patients (*p* = 0.0057, area under the receiver operating characteristic (ROC) curve (AUC) = 0.791, > 12% cluster 8/12-like cells had 77% sensitivity and 74% specificity for ILD); however, TIGIT^+^ cells also performed very well (*p* = 0.0123, AUC = 0.734, > 66% TIGIT^+^ cells had 64% sensitivity and 74% specificity for ILD) (Fig. 4F).

AIC was also associated with loss of CD127 and expression of TIGIT (Fig. 4C). While TIGIT^+^ cells were significant predictors of AIC (*p* = 0.014, AUC = 0.736, > 68% TIGIT^+^ cells had 64% sensitivity and 81% specificity for AIC), TIGIT/CD127 ratio was even more helpful (*p* = 0.009, AUC = 0.751, > 1.5 ratio of TIGIT^+^/CD127^+^ cells had 64% sensitivity and 81% specificity for AIC).

Diarrhea in CVID was not associated with an expansion of the regulatory cluster 16-like cells as it was seen in patients without diarrhea when compared to healthy donors (Fig. 4D). Instead, the expression of activation markers CD38 (*p* = 0.002, AUC = 0.784, > 41% CD38^+^ cells had 71% sensitivity and 84% specificity for diarrhea) and HLA-DR (*p* = 0.006, AUC = 0.753, > 6% HLA-DR^+^ cells had 71% sensitivity and 63% specificity for diarrhea) were both good predictors of this complication.

A similar distribution of cluster 16-like cells and additionally bulk CD8 T cells was seen for the manifestation of hepatopathy (Fig. 1E). The best predictor for hepatopathy in CVID patients was the low proportion of EM1 cells (*p* = 0.028, AUC = 0.763, < 9.4% EM1 cells had 88% sensitivity and 67% specificity for hepatopathy).

These data show a strong association between exhausted phenotype of CD8 T cells, ILD, and AIC and strong association between CD8 T cell activation and non-infectious diarrhea. While the prevalence of CD127^−^PD-1^+^TIGIT^+^CD28^−^-exhausted cluster 8/12-like cells was a strong predictor of ILD, the proportion of TIGIT^+^ cells also performed well in distinguishing such patients.

## Discussion

In this study, we explored the CD8 landscape of patients with CVID and healthy donors, with particular focus on the clinical heterogeneity of CVID as divided into infection only and complex disease groups.

We observed an increase of CD8 T cells in the peripheral blood of CVID patients, shifting from naïve towards more differentiated effector memory and TEMRA stages, in line with findings of earlier studies [14, 15]. However, by combining a higher number of parameters in a single staining analysis, we were able to show that CD27^+^28^−^ and CD27^−^28^−^ effector memory T cells are particularly affected and differ in functional and phenotypic patterns from their CD27^+^28^+^ and CD27^−^28^+^ counterparts. Moreover, our work indicates that accumulation of several distinct populations, including functionally exhausted T cells and activated T cells with immunoregulatory features, represents a major characteristic of CVID and is more pronounced in patients with complex disease.

Through unsupervised exploration of high-parametric dataset obtained with CyTOF, we identified unique cellular clusters upregulated in CVID patients. Chief among these were cell clusters with high functional exhaustion scores; co-expression of inhibitory receptors including PD-1, TIGIT, 2B4, and LAG-3; transcription factors Eomes and TOX; and intermediate expression of cytotoxic and proinflammatory molecules. These are all hallmarks of terminally differentiated exhausted cells [29, 39], which may be a product of an active feedback loop limiting damage in the context of recurrent stimulation with pathogen-derived antigenic stimuli [6, 7] or autoimmunity [40]. A CD127^−^PD-1^+^TIGIT^+^CD28^−^ population corresponding to these exhausted clusters was detectable in the full cohort of 40 patients, upregulated in CVIDc and particularly strongly associated with ILD. The progenitors of exhausted cells which express CXCR5, intermediate levels of PD-1, and the transcription factor TCF1 [41, 42] were preserved in our patients, enabling the maintenance of the terminally exhausted clusters. Patients in our study did not have overt clinical or laboratory signs of ongoing CMV infection, which has been previously shown to drive loss of CD127 and features of CD8 T cell senescence in patients with CVID [17, 43], and ELISpot assessment of CMV-specific T cells performed in a subset of our patients showed evidence of immune memory in only 2/7 of those tested.

The expression of CXCR5 has also been described in recent years in regulatory CD8 T cells and follicular CD8 T cells, first in mice and later in humans [35, 44–46]. While the existence of regulatory CD8 T cells is still contentious in humans and better described in murine models [47], we’ve previously shown the existence of follicular CD8 T cells producing IL-10 in secondary lymphoid organs of patients with CVID and lymphadenopathy [48], which bore transcriptional and phenotypic markers of exhaustion. Here, we see a similar population of CD8 T cells in the peripheral blood, which express relatively high levels of CXCR5, high levels of PD-1, TIGIT, and CTLA-4, lack the IL-7 receptor α subunit CD127, are strongly activated and proliferating, and produce IL-10. Compared to other CD8 T cells, they also have a significantly higher average expression of FoxP3, which is the hallmark of regulatory CD4 T cells [49]. Staining for a corresponding CD127^−^PD-1^high^TIGIT^+^CD28^+^ population in a larger cohort of patients revealed that these cells were not expanded in patients with non-infectious diarrhea and/or hepatopathy as it is seen in patients without these complications. As previously shown, CD21^low^ B cells are associated with autoimmune phenomena [50], and it is thus feasible to speculate on the immunoregulatory role of PD-1^high^CXCR5^+^ CD8 T cells in patients with complex form of CVID, similarly to the regulatory function of the CD44^+^CD122^+^Ly49^+^ CD8 T cells shown in the murine model of autoimmune encephalomyelitis, which also upregulated the *Cxcr5* gene [36].

The diversity of clinical features seen in patients with CVIDc limited the robustness of changes we could note when stratifying cohorts purely based on the division between CVIDio and CVIDc. However, when stratifying patients based on individual complications, more robust patterns emerged. The loss of naïve T cell markers such as CD127 and upregulation of inhibitory receptors, in particular TIGIT, was very strongly associated with autoimmune cytopenia and interstitial lung disease in our cohort and showed significant sensitivity and specificity for history of these particular clinical features. Some data exists to suggest T cell-mediated cytotoxicity as an alternative mechanism for platelet destruction in immune-mediated thrombocytopenia (ITP) [25], and both CD4 and CD8 T cells infiltrate the pulmonary interstitium of patients with CVID [51, 52], but no causal link has yet been shown between CD8 T cell differentiation and ILD or AIC manifestation.

Interestingly, gastrointestinal complaints seen in our patients (non-infectious diarrhea, in some but not all cases accompanied by endoscopic features of enteropathy, with or without villous atrophy) were most strongly associated with CD8 T cell activation, rather than expansion of regulatory CD8 T cells or signs of exhaustion. CD8 T cell activation was previously noted in CVID as Viallard et al. observed an inverse relation between naïve and activated CD8 T cells in CVID [22], Lanio et al. saw higher proportion of activated CD8 T cells in patients with splenomegaly [21], Carter et al. noted elevated proportion of HLA-DR^+^ cells but no association with autoimmune phenomena [19], and de Lollo noted higher proportion of CD127^−^38^+^ cells in patients with splenomegaly compared to healthy donors [20]. Non-infectious chronic diarrhea is a well-described complication of CVID associated with peripheral blood CD8 lymphocytosis, with the majority of intraepithelial lymphocytes seen in CVID enteropathy being CD8^+^ [23], further substantiating the association we observe in the peripheral blood. It’s notable that Han et al. also found an expansion of activated CD38^+^ effector memory CD8 T cells in the intestine of celiac disease patients challenged with gluten, with a corresponding αEβ7 population in the peripheral blood, which were phenotypically similar to the “immunoregulatory” and highly activated cells we found [53], but which were not specifically associated with diarrhea in our cohort. Our own previous work failed to find association between peripheral α4β7 T cell numbers and gastrointestinal disease in CVID but did not evaluate their activation or other phenotype [54]. While infection with norovirus may drive enteropathy in some antibody-deficient patients [55, 56], all but 3 patients we tested were negative for norovirus PCR.

The accumulation of exhausted T cells in complicated CVID observed in this work fits to known mechanisms driving the differentiation of exhausted T cells, such as prolonged antigen stimulation in a chronic inflammatory milieu with inhibitory immune signaling. The specificity of these exhausted T cells in CVIDc, however, is unclear, such as whether they reflect dampened autoimmune responses or insufficient immunity against infectious pathogens, all of which could induce exhausted T cell phenotypes. This raises the question if targeting exhausted T cells could represent therapeutic strategies for symptomatic CVIDc patients. While the latter might potentially underly the expansion of activated T cells associated with infectious diarrhea and could profit from checkpoint therapy, in the context of autoimmune enteropathy and other autoimmune manifestations, such an approach is of high risk of boosting self-reactive T cells and resulting in enhanced autoimmunity. Thus, careful evaluation of the specificity of exhausted T cells in these scenarios is warranted in further studies.

## Conclusion

In summary, in this study, we show that CD8 T cells are highly differentiated, activated, and show an expansion of effector memory populations with signs of increased T cell exhaustion in patients with CVID. These phenotypic changes strongly associate with particular clinical findings such as autoimmune cytopenia, interstitial lung disease, and non-infectious diarrhea, prompting future prospective assessment of T cell phenotype in risk-stratifying patients with respect to screening of non-infectious complications before their clinical manifestation and supporting more aggressive treatment.

We also show a population of activated proliferating CD8 T cells which express CXCR5 and show phenotype and function congruent with their proposed immunoregulatory role. These cells were expanded in CVID patients without gastrointestinal manifestations and previously shown in secondary lymphoid organs of CVID patients. Their biology, ontology, and functions should be explored in more detail, adding to their increasingly acknowledged role in chronic viral infections, autoimmunity, and cancer.

## Supplementary Information

Below is the link to the electronic supplementary material.

Supplementary file1 (DOCX 1893 KB)

## Data Availability

Data is available from authors upon reasonable request. Material is not available.
