# Supplementary material for: Distinct CD8 T Cell Populations with Differential Exhaustion Profiles Associate with Secondary Complications in Common Variable Immunodeficiency
Source: J Clin Immunol. 2022 May 19;42(6):1254–69. doi: 10.1007/s10875-022-01291-9 (PMC9537220; doi:10.1007/s10875-022-01291-9)

**Distinct CD8 T cell populations with differential exhaustion profiles associate with secondary complications in common variable immunodeficiency**

Adam Klocperk, David Friedmann, Alexandra Emilia Schlaak, Susanne Unger, Zuzana Parackova, Sigune Goldacker, Anna Sediva, Bertram Bengsch & Klaus Warnatz

Correspondence:
Adam Klocperk, MD, PhD
Center for Chronic Immunodeficiency (CCI)
Medical Center – University of Freiburg, Faculty of Medicine, University of Freiburg
Breisacher Str. 115
Freiburg im Breisgau
79 106
Germany
ORCID: 0000-0002-1526-4557
Tel: +420 702 013 154
Email: [adam.klocperk@fnmotol.cz](mailto:adam.klocperk@fnmotol.cz)

Table S1 List of antibodies and dyes used in flow cytometric assays

| **Antigen** | **Fluorochrome** | **Clone** | **Company** |
| --- | --- | --- | --- |
| CD3 | BV605 | UCHT1 | BioLegend |
| CD8 | AF647 | SK1 | BioLegend |
| 2B4 | APC-Cy7 | C1.7 | BioLegend |
| PD1 | BV421 | EH12.2H7 | BioLegend |
| CD127 | BV650 | A019D5 | BioLegend |
| TIGIT | PE-Dazzle 594 | A15153G | BioLegend |
| Eomesodermin | PE | WD1928 | BioLegend |
| Tbet | PE-Cy7 | 4B10 | BioLegend |
| CXCR5 | AF488 | RF8B2 | BD |
| CCR7 | PE | 150503 | R&D Systems |
| CD45RA | APC-H7 | HI100 | BD |
| Perforin | BV711 | dG9 | BioLegend |
| CD27 | BV605 | L128 | BD |
| CD28 | PerCP-Cy5.5 | CD28.2 | BioLegend |
| Ki67 | AF700 | Ki-67 | BioLegend |
| CD8 | PE | B9.11 | Beckman Coulter |
| CCR7 | BV650 | G043H7 | BioLegend |
| HLA-DR | BV711 | L243 | BioLegend |
| Granzyme B | BV421 | GB11 | BF Biosciences |
| TCF1 | APC | 7F11A10 | BioLegend |
| CTLA4 | PE | BNI3 | BioLegend |
| CD57 | APC | HCD57 | BioLegend |
| Tim3 | BV711 | F38-2E2 | BioLegend |
| CD8 | PB | SK1 | BioLegend |
| IL-10 | PE | JES3-9D7 | BD |
| Zombie | UV |  | BioLegend |

Table S2 List of antibodies used in mass cytometry

| **Channel** | **Antigen** | **Clone** | **Manufacturer** | **Catalog number** |
| --- | --- | --- | --- | --- |
| 89Y | CD45 | HI30 | Fluidigm | Cat#304002 |
| 104Pd | ß2m-Barcode |  | Henrik Mei |  |
| 105Pd | ß2m-Barcode |  | Henrik Mei |  |
| 106Cd | ß2m-Barcode | 2M2 | BioLegend |  |
| 108Pd | ß2m-Barcode |  | Henrik Mei |  |
| 110Pd | ß2m-Barcode |  | Henrik Mei |  |
| 111 Cd | CD4 | RPA-T4 | BioLegend | Cat#300502 |
| 112 Cd | CD3 | UCHT1 | BioLegend | Cat#300402 |
| 113 Cd | CD39 | A1 | BioLegend | Cat#328202 |
| 114 Cd | IL-17A | BL168 | BioLegend | Cat#512302 |
| 115 In | CD57 | TB01 | invitrogen | Cat#16-0577-85 |
| 116 Cd | CD8 | RPA-T8 | BioLegend | Cat#301002 |
| 139 La | MM-DOTA |  |  |  |
| 140 etc | Beads |  |  |  |
| 141 Pr | CD19 | HIB19 | BioLegend | Cat#302202 |
| 142 Nd | IFNg | B27 | BioLegend | Cat#506502 |
| 143 Nd | GM-CSF | BVD2-21C11 | BioLegend | Cat#502315 |
| 144 Nd | CTLA-4 | BNI3 | BD | Cat#555850 |
| 145 Nd | TNFa | MAb11 | Thermo Fisher | Cat#14-7349-85 |
| 146 Nd | Ki-67 | B56 | BD | Cat#556003 |
| 147 Sm | CD45RA | H100 | BioLegend | Cat#304143 |
| 148 Nd | CD7 | eBio124-1D1 | invitrogen | Cat#14-0079-82 |
| 149 Sm | CD73 | AD2 | BioLegend | Cat#344002 |
| 150 Nd | CD127 | HIL-7R-M21 | BioLegend | Cat#351302 |
| 151 Eu | Granulysin | DH2 | BioLegend | Cat#348008 |
| 152 Sm | IL-2 | MQ1-17H12 | BioLegend | Cat#14-7029-85 |
| 153 Eu | Tim-3 | F38-2E2 | BioLegend | Cat#345002 |
| 154 Sm | XCL-1 | 109001 | R&D | Cat#MAB6951 |
| 155 Gd | CD27 | L128 | BioLegend | Cat#302802 |
| 156 Gd | Helios | 22F6 | BioLegend | Cat#137202 |
| 157 Gd | Foxo1 | 2F8B08 | BioLegend | Cat#658102 |
| 158 Gd | PD-1 | EH12.2H7 | BioLegend | Cat#329902 |
| 159 Tb | CCR7 | G043H7 | BioLegend | Cat#353202 |
| 160 Gd | Tbet | 4B10 | BioLegend | Cat#644802 |
| 161 Dy | CD28 | CD28.2 | BioLegend | Cat#302902 |
| 162 Dy | FoxP3 | PCH101 | invitrogen | Cat#14-4776-82 |
| 163 Dy | TCF1 | 7F11A10 | BioLegend | Cat#655202 |
| 164 Dy | CXCL10 | J034D6 | BioLegend | Cat#519502 |
| 165 Ho | EOMES | WD1928 | Thermo Fisher | Cat#14-4877-82 |
| 166 Er | Perforin | B-D48 | abcam | Cat#ab47225 |
| 167 Er | CD38 | HIT2 | Thermo Fisher | Cat#14-0389-82 |
| 168 Er | TOX | Rea473 | Miltenyi | Cat#130-118-335 |
| 169 Tm | TIGIT | MBSA43 | Thermo Fisher | Cat#16-9500-82 |
| 170 Er | CXCR5 | RF8B2 | BD | Cat#552032 |
| 171 Yb | 2B4 | C1.7 | BioLegend | Cat#329502 |
| 172 Yb | CD160 | BY55 | BioLegend | Cat#341202 |
| 173 Yb | HLA-DR | L243 | BioLegend | Cat#307602 |
| 174 Yb | IL-10 | JES3-9D7 | BioLegend | Cat#501402 |
| 175 Lu | LAG-3 | 17B4 | enzo | Cat#ALX-804-806-C100 |
| 176 Yb | CD120b | 3G7A02 | BioLegend | Cat#358402 |
| 191/193 | Iridium |  |  |  |
| 198 Pt | ß2m-Barcode | 2M2 | Biolegend |  |
| 209 Bi | CD16 | 3G8 | Fluidigm | Cat#3165001B |

**Fig. S1** Gating strategy for memory subsets, CD127, TIGIT and PD1 markers assessed by flow cytometry


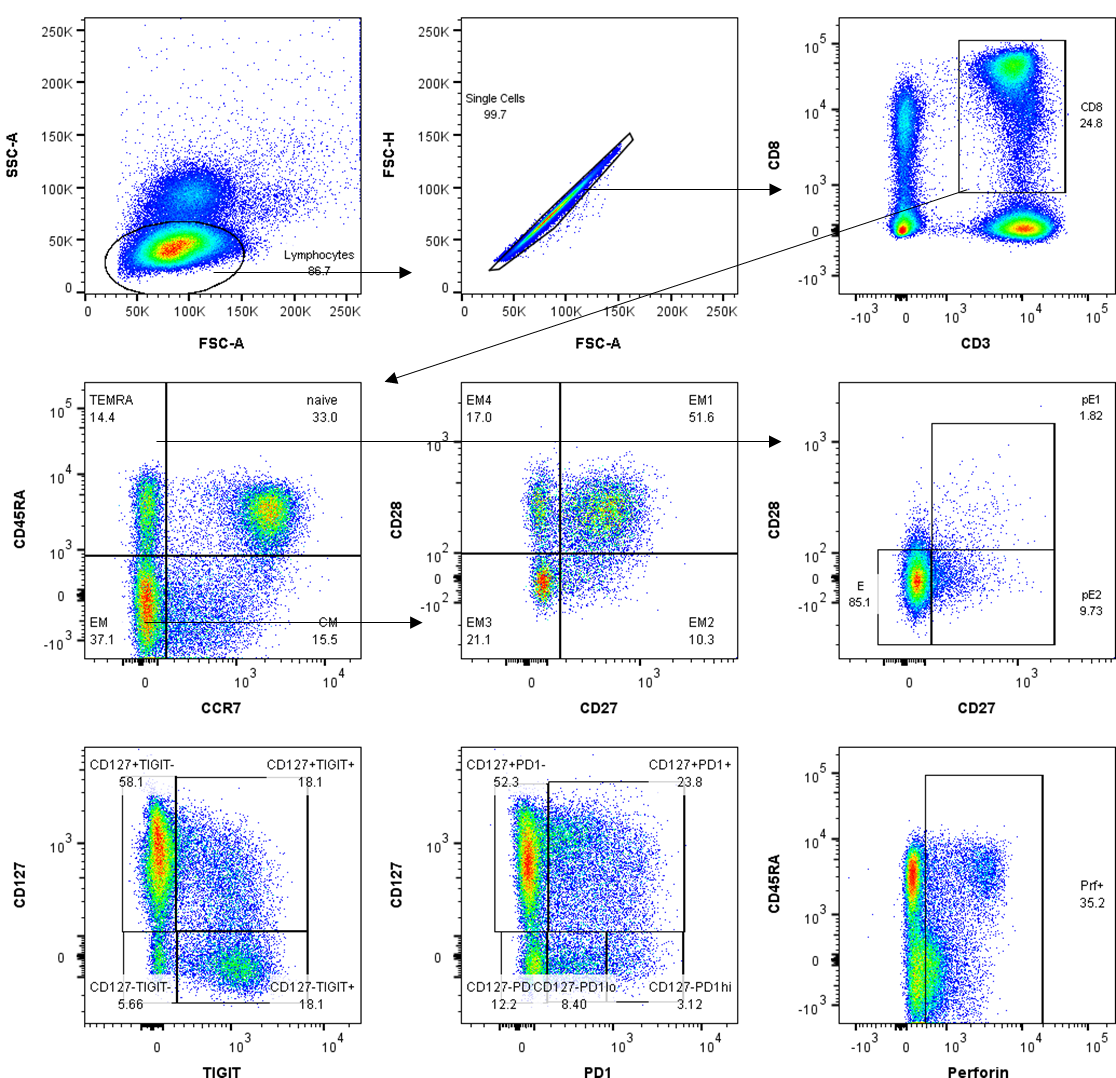


**Fig. S2** CD4:8 T cell ratio in HD, CVIDio and CVIDc (n = 57, 17 HD, 11 CVIDio, 29 CVIDc) as assessed by flow cytometry

**Fig. S3** IL-2 and perforin staining of CD4 T cells (top row), EM2 CD8 T cells (middle row) and EM3 CD8 T cells (bottom row) as assessed by CyTOF (n = 12, 4 HD, 4 CVIDio, 4 CVIDc)


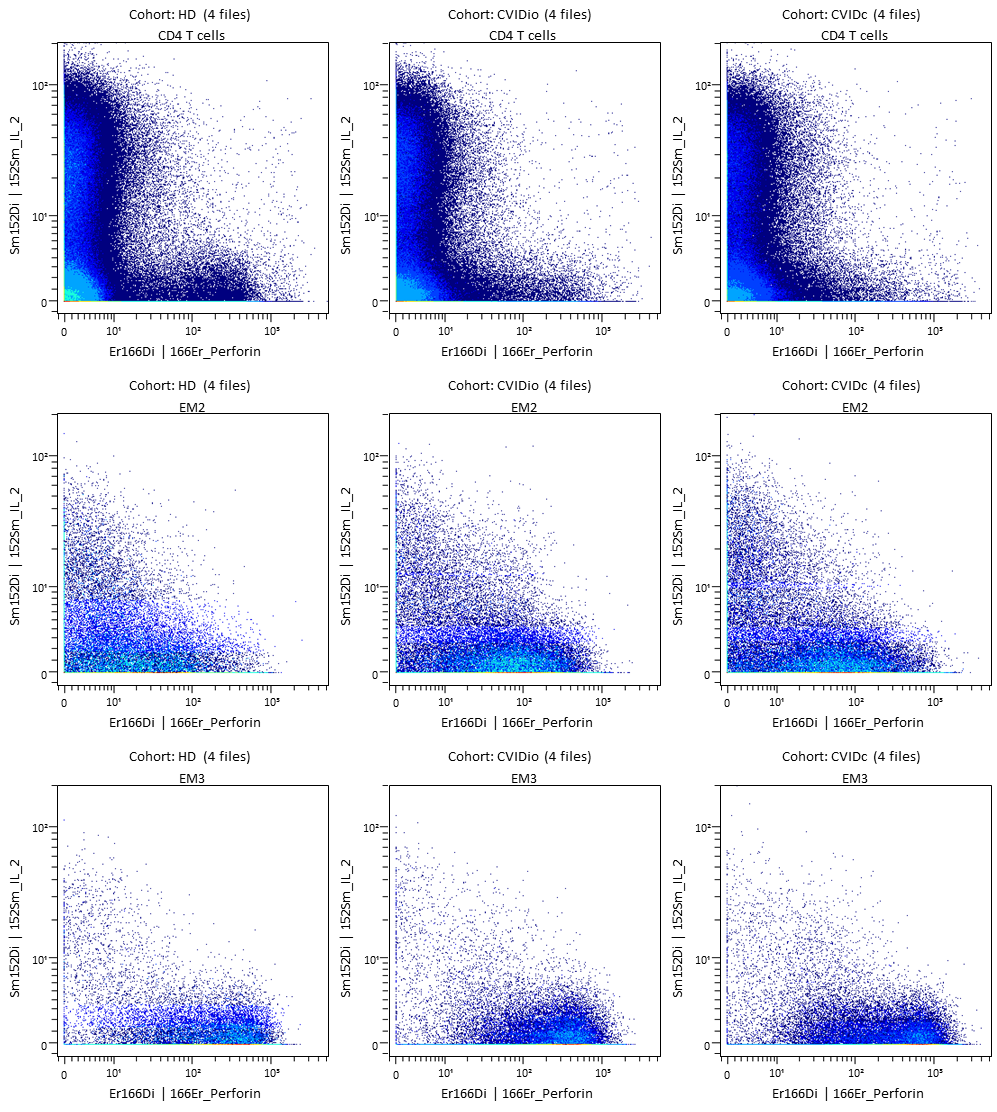


**Fig. S4** CyTOF-derived cluster proportion in all samples, as % of CD8 T cells (n = 12, 4 HD, 4 CVIDio, 4 CVIDc)


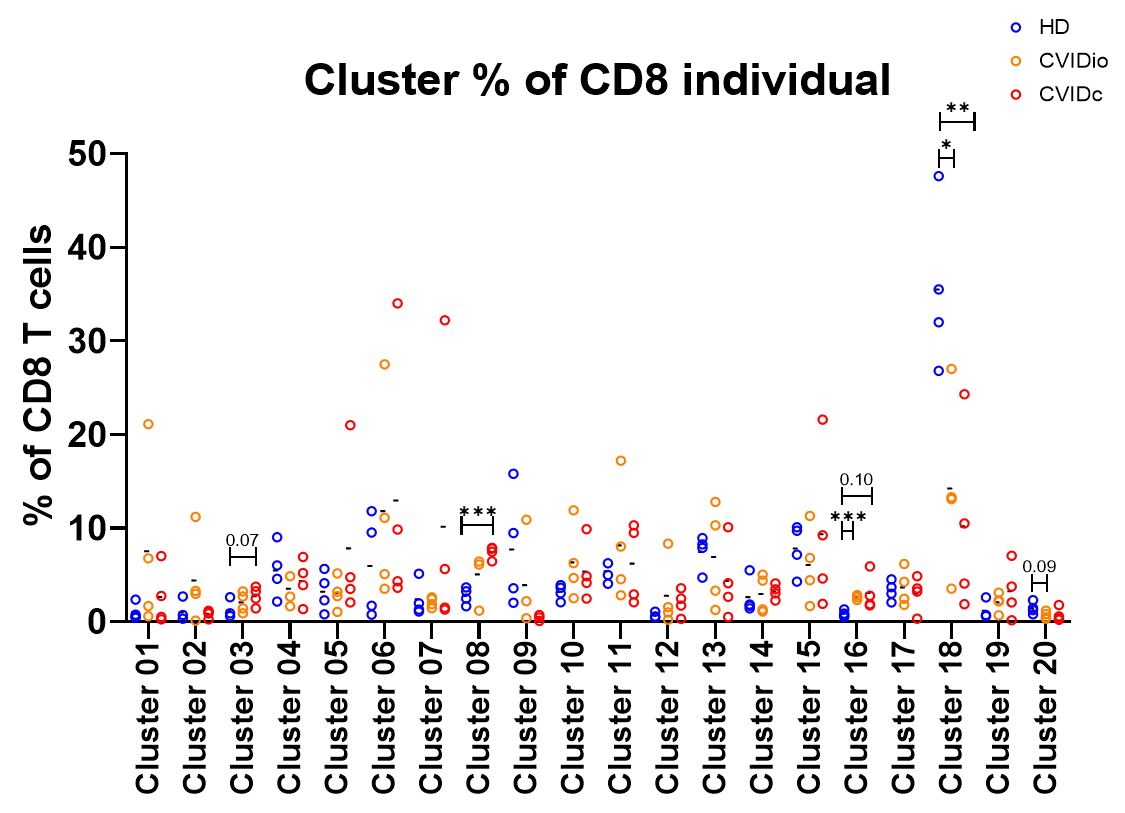


**Fig. S5** FES score of all samples within each CyTOF-derived cluster (n = 12, 4 HD, 4 CVIDio, 4 CVIDc)


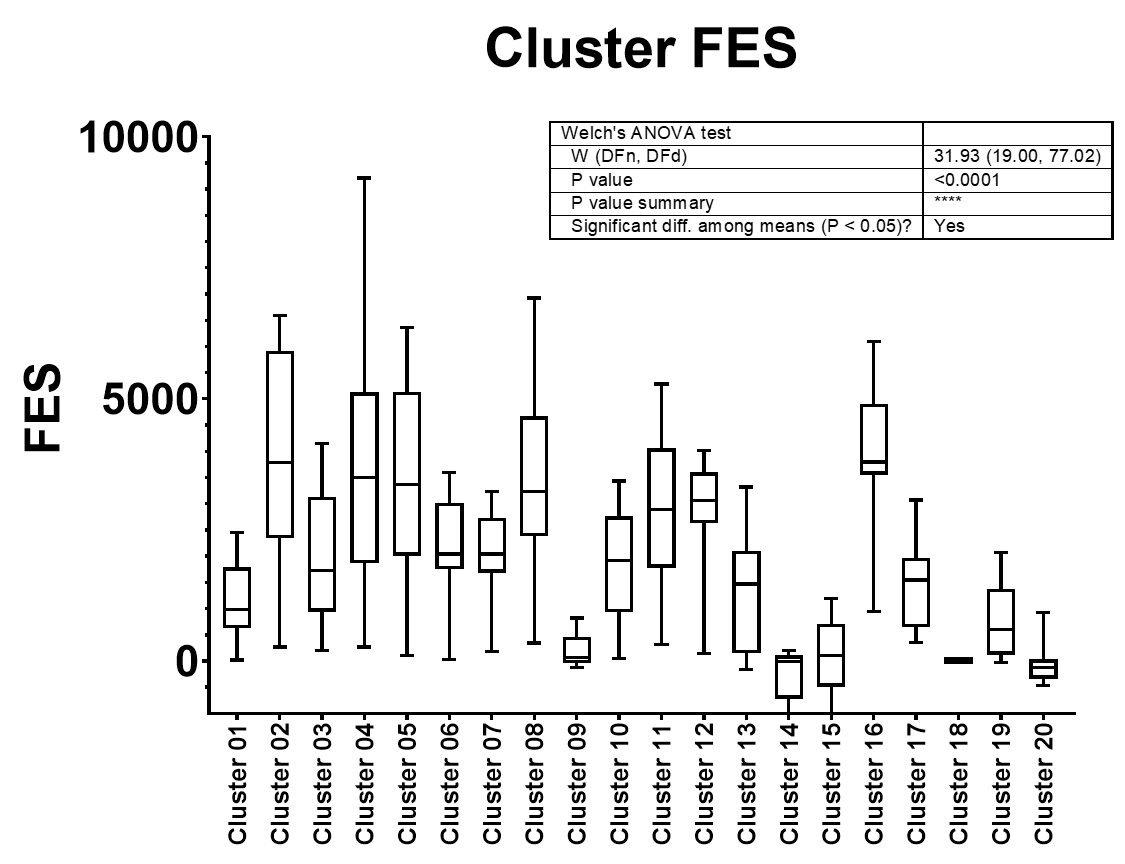


**Fig. S6** Expression of PD1, IL-10, CD127 and FoxP3 in CD8 clusters 13, 16, 18 and bulk CD4 T cells as assessed by CyTOF (n = 12, 4 HD, 4 CVIDio, 4 CVIDc)


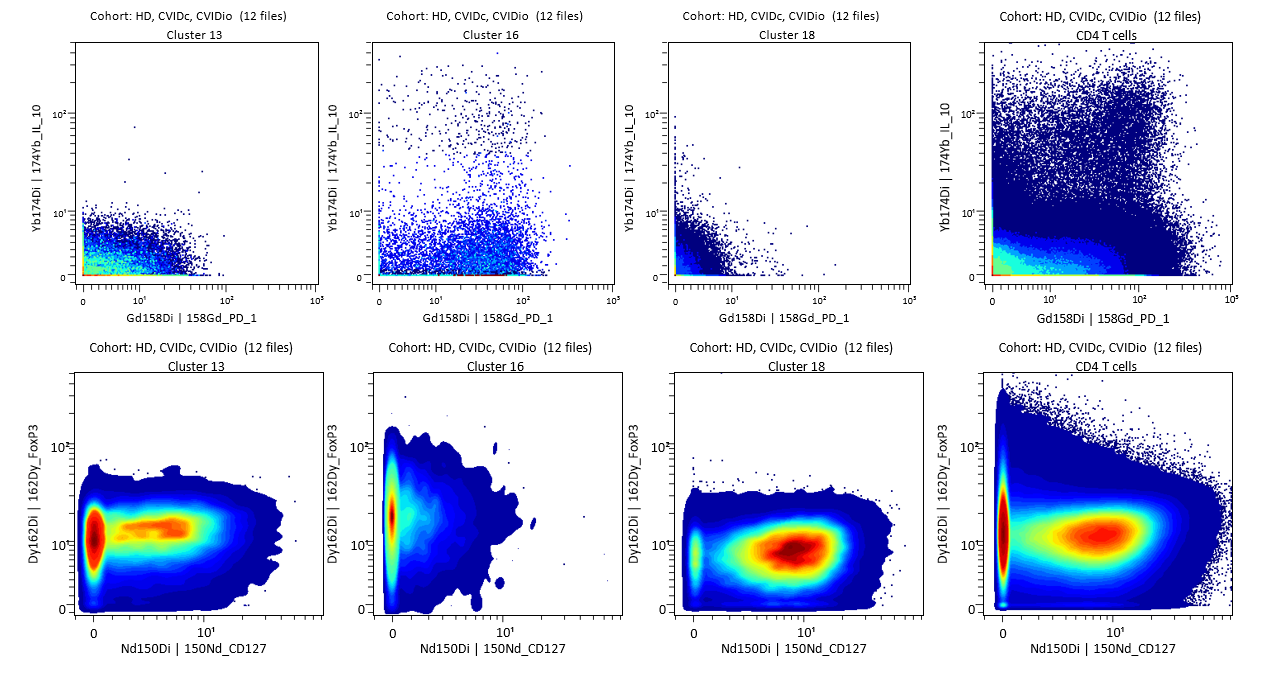


**Fig. S7** Expression of markers in all samples stained with flow cytometry (n = 57, 17 HD, 11 CVIDio, 29 CVIDc). Color scale shows Z-score, missing values are white with a cross-through

**Fig. S8** Expression of PD1, CD127 and TIGIT in naive, EM, EM3, TEMRA and CD27-28- TEMRA CD8 T cells assessed by flow cytometry (n = 57, 17 HD, 11 CVIDio, 29 CVIDc)


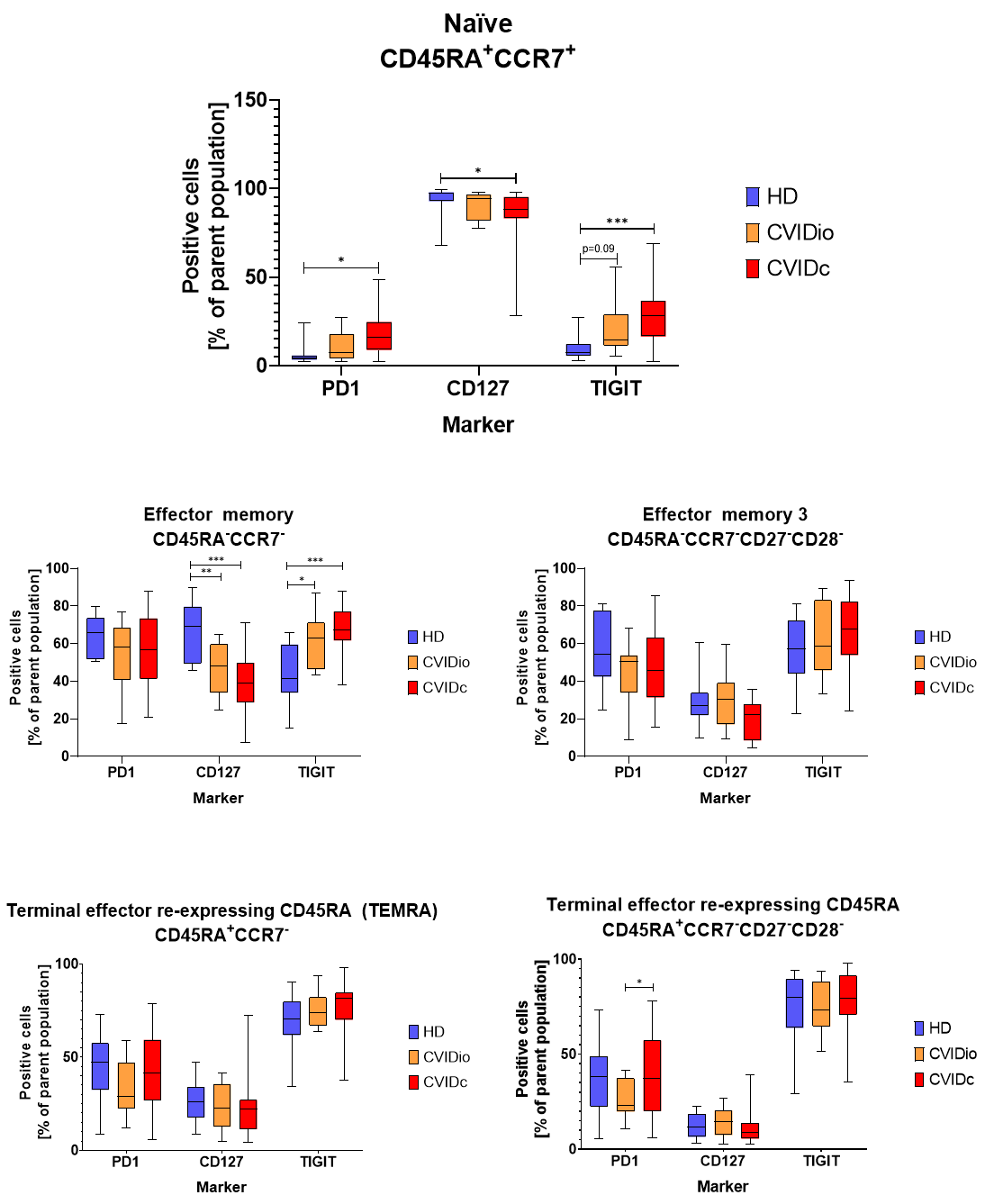

Supplement: Supplementary file 1 — Supplementary file1 (DOCX 1893 KB) [file 10875_2022_1291_MOESM1_ESM.docx]
